# Developing countries have had enough: Is it time for a tobacco endgame?

**DOI:** 10.18332/tid/115066

**Published:** 2020-01-07

**Authors:** Joshua Kibet, Bornes C. Mosonik

**Affiliations:** 1 Department of Chemistry, Egerton University, Njoro, Kenya

**Keywords:** addiction, tobacco, cessation

**Dear Editor,**

Extensive research has been published on the dangers of tobacco and the miseries it has caused to human health and economies, yet its consumption appears to have gained significant momentum^1,2^, with addiction, illness and poverty among the major outcomes. Extensive funding has been allocated to combat the tobacco epidemic with little progress, especially in developing countries. It appears, therefore, that the time has come to confront tobacco companies head-on in order for them to explain the philosophy behind tobacco business at the expense of human life. This would be one of the most radical decisive measures in addition to classifying tobacco as a hard drug — in the same group with heroin, cannabis, and cocaine. This has been supported before by other researchers as one of the most effective methods towards achieving tobacco endgame. For instance, banning tobacco products has reduced smoking risk among the youth and as such, strong e-cigarette regulation measures are required to achieve cessation^3^. Enacting legislation to ban tobacco, considering the present legal status it enjoys, is not easy but governments across the globe should be encouraged to pursue this route.

Consequently, persons or companies dealing with tobacco business or even promoting the growing of tobacco should be punishable by law through fines, jail terms or both. At the heart of this concern are the psychological, emotional, addiction and economic burdens induced by tobacco misuse^1,4^. It should therefore be noted that although tobacco leaf generates significant revenue to most economies, the cost of tobacco related ailments is painfully high^5^. The introduction of e-cigarettes into the market was aimed at promoting tobacco cessation but its dual-use by both smokers and non-smokers has dampened its impact as a potential cessation strategy^6^. Moreover, there have been reports that increases in vaping (e-cigarette use) have been associated with declining rates of youth smoking^7^. From a research standpoint, e-cigarettes are not meant for people who have never smoked in their life, and even so, their efficacy as a smoking cessation device needs thorough and long-term research, although cases of pulmonary related outcomes have recently been reported^8,9^. This is a grave concern to both medical personnel and public health authorities in their campaign against tobacco abuse.

In summary, as long as tobacco companies are allowed to operate tobacco businesses as usual, no amount of campaigning against tobacco abuse will succeed effectively. It should be noted clearly that tobacco producing companies (TPCs) have now shifted focus to developing countries in what is dubbed as an economic charm offensive^10,11^. Sub-Saharan countries have lately welcomed TPCs and are now the greatest producers of tobacco leaf^12^. With the changing face of tobacco and nicotine products and the activities of TPCs, achieving tobacco endgame is a herculean task, nevertheless it should be an ultimate goal.
